# Understanding missed nursing care through felt accountability focus and task complexity: A prospective repeated-measures field study

**DOI:** 10.1016/j.ijnsa.2025.100447

**Published:** 2025-11-10

**Authors:** Layla Suliman, Anat Drach- Zahavy, Hadass Goldblatt, Hanna Admi, Ilana Peterfreund, Liora Sabah, Einav Srulovici

**Affiliations:** a The Cheryl Spencer Department of Nursing, University of Haifa, Haifa, Israel; b Ziv Nursing School, Zefat, Israel; c The Max Stern Yezreel Valley College, Afula, Israel; d Bnai Zion Medical Center, Haifa, Israel

**Keywords:** Missed nursing care, Accountability focus, Task complexity, Patient safety, Quality of care

## Abstract

**Background:**

Missed nursing care is a key indicator of compromised care quality, often attributed to workload and staffing. Yet it may also be influenced by accountability focus—whether nurses are evaluated on outcomes or on processes. Evidence suggests that its effect on performance, and thus on missed care, may depend on task complexity, but this has not been tested in clinical practice

**Aim:**

To investigate whether task complexity (simple vs. complex) moderates the association between nurses’ felt accountability focus (outcome vs. process) and missed nursing care.

**Design:**

A prospective repeated-measures field study with nurses nested within wards.

**Setting:**

The study was conducted in one medium-sized public hospital, encompassing 10 internal medicine and surgical wards.

**Participants:**

A total of 105 registered nurses providing direct patient care participated in the study.

**Methods:**

Data were collected between November 2023 and April 2024. Each nurse completed anonymous mobile survey at the end of three different shifts, yielding 315 repeated observations nested within 105 nurses. Nurses completed questionnaires assessing missed nursing care, task complexity, felt accountability focus, and sociodemographic characteristics. Mixed linear modeling accounted for the repeated-measures design and nested data structure.

**Results:**

No direct effects of task complexity and felt accountability focus were found. However, both interaction effects were statistically significant: task complexity × felt process accountability focus (β = −0.04, *p* = 0.012) and task complexity × felt outcome accountability focus (β = 0.04, *p* = 0.006). Probing revealed a crossover pattern: under simple tasks, higher outcome accountability was associated with less missed care, whereas under complex tasks, higher process accountability was associated with less missed care.

**Conclusion:**

Accountability should be adapted to task complexity rather than applied uniformly. Outcome accountability enhances consistency in routine care, whereas process accountability safeguards thoroughness in complex situations. These findings extend accountability research into real-world nursing practice and offer actionable guidance for designing systems that promote safe, high-quality care.

**Social media abstract:**

Accountability focus impacts missed nursing care differently by task complexity. This field study bridges lab findings & clinical practice, offering a framework to design smarter accountability systems that protect patients & support nurses. #NursingResearch #PatientSafety #HealthcareQuality #NurseLeadership

**What is already known?**

• Missed nursing care is a widespread indicator of compromised care quality, usually attributed to structural factors such as workload and staffing.
• Accountability focus, distinguishing between outcome- and process-based evaluation, is an organizational mechanism that can shape employee performance.
• Prior evidence on the relative benefits of process versus outcome accountability is mixed and comes mainly from experimental or non-clinical settings.

Alt-text: Unlabelled box dummy alt text

**What this paper adds?**

• The study demonstrates that accountability focus alone does not directly predict missed nursing care in clinical settings.
• It shows that the effectiveness of accountability depends on task complexity: outcome accountability reduces missed care in simple tasks, whereas process accountability reduces missed care in complex tasks.
• It provides actionable guidance for developing accountability frameworks aligned with clinical task demands to improve patient safety and nurse well-being.

Alt-text: Unlabelled box dummy alt text

## 1 Background

### 1.1 The burden and consequences of missed nursing care

Nurses operate at the frontline of increasingly strained healthcare systems, where patient acuity is rising, staffing is often inadequate, and time-critical decisions must be made under persistent pressure (Juvé-Udina et al., 2020; Stephenson et al., 2024). In such environments, the ability to deliver safe, complete, and timely care is often compromised. One outcome of these conditions is missed nursing care—defined as any required patient care that is omitted, delayed, or incompletely delivered (Kalisch et al., 2009). Missed care is widely recognized as a key indicator of compromised care quality, reflecting the tension between system-level constraints and nurses’ professional responsibilities (Abdelhadi et al., 2020; Khajoei et al., 2025; Srulovici and Drach-Zahavy, 2017). Evidence shows it is both prevalent and consequential: a recent meta-analysis found that 80 % of nurses reported missing at least one care activity during their most recent shift (Labrague and Cayaban, 2025). Such omissions have been linked to poorer patient outcomes, reduced nurse well-being, and negative organizational effects (Chaboyer et al., 2021; Lake et al., 2019).

### 1.2 Antecedents of missed nursing care: structural, personal, and organizational factors

Research has highlighted multiple layers of antecedents to missed nursing care. At the structural level, factors such as excessive workload, understaffing, and time pressure remain central (Griffiths et al., 2018). However, growing attention has been directed toward personal, contextual, and organizational influences. At the personal level, studies have shown that nurses’ internal sense of accountability—defined as the willingness to take ownership, act transparently, and be answerable for one’s clinical decisions—is inversely associated with missed care (Cohen et al., 2024; Srulovici and Drach-Zahavy, 2017). Contextual factors, such as high patient acuity or challenging behaviors, also increase the likelihood of missed care (Abdelhadi et al., 2022, 2023; Chaboyer et al., 2021; Lake et al., 2020b). At the organizational level, leadership style and team functioning—such as proactive head nurse behaviors and strong teamwork—have been found to reduce missed nursing care (Chaboyer et al., 2021; Lake et al., 2020a; Nobahar et al., 2023). These findings align with recent evidence underscoring the importance of fostering a strong patient safety culture as a strategic means to minimize missed nursing care (Labrague and Cayaban, 2025).

### 1.3 Accountability as an organizational mechanism

Although personal characteristics tend to be stable and contextual conditions often lie beyond the nurse’s control, organizational characteristics offer a more flexible and actionable target for intervention. One such characteristic is accountability focus—whether employees are evaluated based on the outcomes they achieve or the processes they follow (Hall et al., 2017). Outcome accountability emphasizes end results, often reflected in quality indicators (Foulkes, 2011), whereas process accountability emphasizes adherence to procedures and ethical reasoning (Bail and Grealish, 2016). Scholars have argued that process accountability is preferable because it fosters autonomy, deliberate reasoning, and fairness in performance evaluations, particularly when outcomes are influenced by external factors (Hall et al., 2017; Scholten et al., 2007). In contrast, outcome accountability may lead to stress, poor communication, and lower performance when individuals are judged by outcomes beyond their control (Hall et al., 2007; Siegel-Jacobs and Yates, 1996).

### 1.4 Task complexity as a moderating factor in performance

Empirical findings on the superiority of process accountability are mixed. While some individual studies have suggested that process accountability improves decision-making and ethical behavior (Scholten et al., 2007; Sharon et al., 2022; Siegel-Jacobs and Yates, 1996), others have found benefits for outcome accountability in terms of efficiency and innovation (Langhe et al., 2011; Sharon et al., 2022). To resolve these discrepancies, a recent meta-analysis examined the direct relationship between accountability focus and performance and found no consistent main effect (Sharon et al., 2022). This absence of a direct link suggests that accountability focus alone does not uniformly influence performance. Instead, the meta-analysis concluded that the effectiveness of accountability focus depends on task complexity, which can act as a moderator.

Task complexity refers to the degree of cognitive effort required to complete a task, and is shaped by three dimensions: component complexity (number of cues and actions), coordination complexity (interdependence among components), and dynamic complexity (degree of required adaptation) (Wood, 1986). In nursing practice, tasks vary widely in complexity, encompassing responsibilities such as data collection, diagnosis, care planning, patient education, and clinical decision-making (Stokes and Palmer, 2020). Nurses must therefore be prepared to manage tasks across the complexity spectrum to ensure safe, high-quality care. For instance, conducting a foot evaluation for patients with diabetes—a common task aimed at preventing complications such as amputation (CDC, 2024) —may appear simple when guidelines from the Ministry of Health or the American Diabetes Association (ADA) are followed. However, the task becomes more complex when risk factors or ulcers are present. This increases component complexity (e.g., additional steps such as wound assessment), coordination complexity (e.g., involving physicians for referral or imaging), and dynamic complexity (e.g., responding to changes in the patient’s physical or mental condition) (ADA, 2021). In such scenarios, the same task requires greater cognitive effort and resource investment, highlighting the context-dependent and variable nature of task complexity in nursing. Accordingly, it becomes crucial to examine accountability focus not as a static organizational mandate, but as it is experienced (i.e., felt accountability focus) and interpreted by the practicing nurse in the face of real-time clinical demands.

### 1.5 Gaps in the literature and the need for a field study

There is growing evidence that task complexity statistically significantly influences human performance and may alter the effects of accountability focus (Liu and Li, 2012). International research has also shown that missed nursing care is particularly prevalent in internal medicine and surgical wards, where workload and task complexity are high (Chaboyer et al., 2021; Vexler et al., 2025) .Experimental research has reported conflicting patterns, with some studies finding process accountability improves analytical performance in simple tasks while others suggesting outcome accountability may enhance performance in complex contexts (Langhe et al., 2011; Siegel-Jacobs and Yates, 1996). A recent meta-analysis confirmed that task complexity moderates the link between accountability focus and performance, yet revealed that nearly all available evidence originates from laboratory settings and non-clinical samples (Sharon et al., 2022). Only a few nursing studies have indirectly addressed accountability—for example, as a moral or organizational attribute associated with reduced missed nursing care (Abdelhadi et al., 2020; Cohen et al., 2024; Leonenko and Drach-Zahavy, 2016; Srulovici and Drach-Zahavy, 2017) or as part of simulation-based designs testing theoretical models (Sharon et al., 2024). While these studies advanced conceptual understanding, none examined how felt accountability focus interacts with the real-world complexity of nursing tasks to predict care performance. Consequently, the empirical foundation linking accountability focus, task complexity, and missed nursing care in authentic clinical environments remains underdeveloped. The present study directly addresses this gap by conducting, to our knowledge, the first in-vivo field investigation of how nurses’ felt process versus outcome accountability focus interacts with task complexity to influence missed nursing care, thereby bridging experimental and organizational lines of inquiry.

### 1.6 Conceptual and theoretical framework

**Fig. 1:**
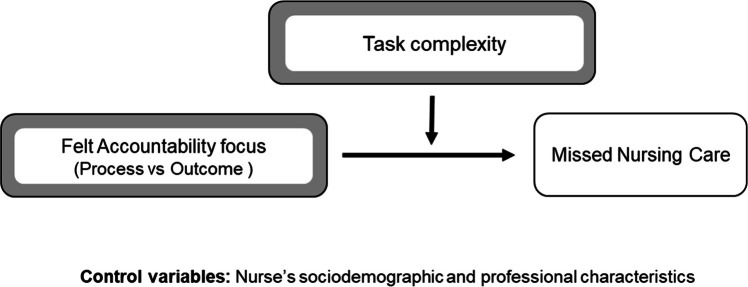
Research model.

The study introduces a person-in-context moderation model (Fig. 1), grounded in two complementary theoretical frameworks. The first is the accountability framework (Frink and Klimoski, 2004), which conceptualizes accountability as a relational mechanism through which individuals anticipate being evaluated and adjust their behavior accordingly (Hall et al., 2017; Hochwarter et al., 2007). The second is the resource allocation model (Kanfer and Ackerman, 1989), which posits that individuals possess limited cognitive and emotional resources that must be allocated based on task demands. According to this integrated model, task complexity moderates the relationship between felt accountability focus and performance by influencing the cognitive load required to complete tasks. Process accountability, by fostering deliberate and reflective engagement, may help reduce strain and improve performance on complex tasks. Conversely, outcome accountability may be more effective for simpler tasks that demand rapid, results-oriented action without excessive cognitive investment (Häusser et al., 2017). By situating accountability focus within the varying demands of nursing tasks, this study addresses an important gap in understanding when and how accountability frameworks improve or undermine clinical performance.

### 1.7 Research aim

The aim of this study was to examine how nurses’ felt accountability focus (outcome vs. process) interacts with task complexity (simple vs. complex) to influence the frequency of missed nursing care in acute care settings.

### 1.8 Research hypotheses

H1: No significant differences will be found in missed nursing care between felt accountability focuses.
H2: The interaction between felt accountability focus and task complexity will be associated with a reduced frequency of missed nursing care among nurses, such that:
(a) Under complex tasks, nurses reporting higher felt process accountability focus will be associated with a reduced frequency of missed nursing care than those with lower felt process accountability focus.
(b) Under simpler tasks, nurses reporting higher felt outcome accountability focus will be associated with a reduced frequency of missed nursing care than those with lower felt outcome accountability focus.

## 2 Methods

### 2.1 Design and sample

This study employed a prospective repeated-measures design involving registered nurses from 10 internal medicine and surgical wards at a medium-sized central hospital in Israel. All internal medicine and surgical wards in the hospital were included, as they represent the largest inpatient units with high patient turnover and thus provide a suitable context for examining missed nursing care. These departments have also been shown in previous studies to experience some of the highest rates of missed care due to workload and task complexity (Chaboyer et al., 2021; Vexler et al., 2025). Intensive care units were excluded because their unique staffing models, nurse-to-patient ratios, and continuous monitoring systems make missed care conceptually and empirically less comparable to general wards.

All nurses working in the selected wards were invited to participate. Inclusion criteria were: (a) being a front-line registered nurse, (b) having at least six months of tenure in the ward, and (c) working at a minimum of 75 % full-time equivalency (FTE). Nurses who did not meet these criteria were not included in the study. Additionally, the exclusion criteria included: (a) nurse managers, educators, or administrative staff without direct patient care responsibilities, (b) temporary or float nurses not permanently assigned to the participating wards, and (c) nurses on extended leave during the data collection period.

A priori power analysis using G*Power software was conducted to determine the minimum required sample size for repeated-measures ANOVA for between-subject factors. Assuming a medium effect size (*f* = 0.25) based on Cohen's (1988) conventions for behavioral and social sciences, which have been widely adopted in nursing research, α = 0.05, power = 0.80, number of measurements = 3, and correlation among repeated measures = 0.5, the analysis indicated that at least 86 participants were needed. Of the 135 nurses who consented, 105 completed all three measurement points and were included in the final analysis.

### 2.2 Measures

All instruments used in this study were previously validated and widely applied in nursing and organizational research. Reliability coefficients (Cronbach’s α) for each measure in the current sample are reported below.

#### 2.2.1 Dependent variable - missed nursing care

Missed nursing care was assessed using the 22-items MISSCARE survey (Kalisch et al., 2009), which asks nurses to rate the frequency of missed care activities in the last shift on a 4-point Likert scale (1 = rarely missed, 4 = always missed). Items marked as “not applicable” (coded as 9) were treated as missing values in SPSS and excluded from score calculations. Example items include “Emotional support to patient and/or family” or “Response to call light is initiated within 5 min”. The score was calculated as the average across the 22 items, with higher scores indicating more frequent missed care (Cronbach’s alpha 0.95). Internal consistency in this study was high (Cronbach’s α =0.91–0.98).

#### 2.2.2 Independent variable - felt accountability focus

Felt accountability focus was assessed via two items adapted from (Brtek and Motowidlo, 2002). The first question was designed to assess felt outcome accountability focus: “Think about the outcomes of performing your tasks. While carrying out these tasks, to what extent did you believe you might need to explain to someone else (e.g., head nurse, etc.) why there are differences between your performance results and those of nursing experts?”. The second question was designed to assess felt process accountability focus: “Think about the decision-making process in performing your tasks. To what extent did you believe you might need to explain to someone else (e.g., head nurse, etc.) your decision-making”. Responses were rated on a 7-point Likert scale (1 = definitely did not believe I would have to explain, 7 = definitely believed I would have to explain).

#### 2.2.3 Moderation variable –task complexity

Task complexity was assessed using a 4-items subscale from the Work Design Questionnaire (Morgeson and Humphrey, 2006). Participants rated the complexity of tasks performed during the last shift on a 5-point Likert scale (1 = strongly disagree to 5 = strongly agree). An example item is: “The tasks I performed during my last shift were simple and not complex”. All items were reverse coded, and an overall task complexity score was derived by calculating the mean of the four subscales. Higher scores indicate greater task complexity, whereas lower scores reflect simpler tasks. (Cronbach’s α = 0.81–0.88).

#### 2.2.4 Control variables

##### 2.2.4.1 Workload

Workload in the last shift was assessed using the National Aeronautics and Space Administration (NASA) Task Load Index (Hart and Staveland, 1988), which evaluates six dimensions of perceived workload: mental demand, physical demand, temporal demand, performance, effort, and frustration. Each was rated on a 20-point scale (1 = low, 20 = high). The overall workload score was calculated as the average of the six subscales. An example item is: “How much mental and perceptual activity was required—thinking, deciding, calculating, searching? Was the shift easy or demanding, simple or complex?” (Cronbach’s α = 0.72). Internal consistency in this study was α = 0.81–0.88.

##### 2.2.4.2 Sociodemographic and professional characteristics

Participants reported their age, gender (male, female), educational level (registered nurse without a bachelor’s degree, with a bachelor’s degree, or with a postgraduate degree), professional nursing role (head nurse, clinical instructor, deputy head nurse, bedside nurse), years of experience as a nurse and within the ward, and percentage of FTE employment.

### 2.3 Data analysis

Descriptive statistics were computed for all study variables, including means, standard deviations, and frequencies where appropriate. Pearson correlations were conducted to examine bivariate relationships among the main variables. To test the study hypotheses, a linear mixed-effects model was employed to account for the repeated-measures design, with three observations nested within each nurse. The dependent variable was missed nursing care. Fixed effects included felt process and outcome accountability focuses, task complexity, and their interaction terms. Only control variables that showed significant correlations with the dependent variable were included in the model. Mixed linear model assumptions (normality, homoscedasticity, and independence of residuals) were verified through visual inspection of Q–Q plots and residuals versus fitted values plots and were met. All analyses were conducted using SPSS version 27, with statistical significance set at *p* < 0.05.

### 2.4 Data collection and ethical considerations

Ethical approval for this study was obtained from the institutional review board of the Medical Center (Approval No. BNZ-0029–23, date: 17 August 2023), as well as the Ethics Committee of The Faculty of Social Welfare and Health Sciences at the University (Approval No. 234/23, date: 26 June 2023), before commencement of the study. The research team met with head nurses to explain the study’s objectives and procedures. Subsequently, participating nurses were briefed during staff meetings and provided informed consent, with the assurance that participation was voluntary and withdrawal could occur at any time without consequences.

Data collection occurred between November 2023 and April 2024. At the end of three different shifts, typically spaced one to two weeks apart depending on individual shift schedules, nurses received an anonymous Qualtrics survey link via mobile phone. The survey was programmed to require a response for each item before submission, ensuring complete data for all participants. Consequently, no missing values were detected across study variables. To enable linkage across time points while maintaining anonymity, participants entered a self-generated code. All data were stored on a secure university network.

## 3 Results

### 3.1 Descriptive statistics

The final sample consisted of 105 registered nurses across 10 hospital wards (range = 3–15 nurses per ward, mean = 10 nurses per ward). Table 1 presents the descriptive statistics and bivariate correlations for all study variables. Participants ranged in age from 22 to 60 years (*M* = 35.61, SD = 8.45), with most identifying as female (*n* = 79, 75.1 %) and holding a bachelor’s degree in nursing (*n* = 78, 75.4 %). Their professional experience spanned from 1 to 41 years (*M* = 8.85, SD = 8.42).

**Table 1:** Descriptive statistic and correlations between study variables.

	Characteristics	Mean	(SD)	1	2	3	4	5	6	7
1	Missed nursing care	1.42	(0.38)	1						
2	Age	35.61	(8.45)	0.00	1					
3	Seniority in nursing	8.85	(8.42)	−0.07	0.82⁎⁎	1				
4	Workload	10.03	(4.05)	0.48⁎⁎	0.03	−0.05	1			
5	Task complexity	3.40	(1.06)	0.33⁎⁎	0.07	0.03	0.60⁎⁎	1		
6	Felt outcome accountability focus	4.53	(1.45)	0.07	−0.04	−0.11*	0.17⁎⁎	0.12*	1	
7	Felt process accountability focus	5.14	(1.25)	−0.02	0.08	−0.01	0.07	0.13*	0.58⁎⁎	1

Note: SD, standard deviation.

⁎ *p* < 0.05.

⁎⁎ *p* < 0.01.

Across three measured shifts, nurses reported occasional levels of missed nursing care (*M* = 1.42, SD = 0.38). Felt process accountability focus had a mean score of 5.14 (SD = 1.26), while felt outcome accountability focus averaged 4.53 (SD = 1.45). The average perceived workload was moderate (*M* = 10.04, SD = 4.05), and perceived task complexity was relatively moderate (*M* = 3.40, SD = 1.06).

### 3.2 Correlation findings

Bivariate correlations (Table 1) revealed a statistically significant positive relationship between task complexity and missed nursing care (*r* = 0.33, *p* < 0.001). Moreover, professional nursing role was statistically significantly associated with missed nursing care (F (2354) = 3.31, *p* = 0.038). However, neither felt process accountability focus nor felt outcome accountability focus was statistically significantly correlated with missed nursing care (*p* > 0.05).

### 3.3 Hypotheses testing

To examine the hypothesized moderation effects, a three-step mixed linear model was conducted with missed nursing care as the dependent variable (Table 2). In Step 1, control variables that were statistically significantly associated with missed care in the correlation analysis were entered. Of these, only workload was a statistically significant predictor (β = 0.04, *p* < 0.001).

In Step 2, the main effects of task complexity, felt process accountability focus, and felt outcome accountability focus were entered. None of these variables were statistically significantly associated with missed nursing care: task complexity (*p* = 0.098), felt outcome accountability focus (*p* = 0.743), and felt process accountability focus (*p* = 0.605). These findings support Hypothesis 1, which proposed no statistically significant main effects.
In Step 3, the interaction terms between task complexity and each type of felt accountability focus were added. The fixed effects collectively explained 18.8 % (η² = 0.18) of the variance in missed nursing care, indicating a medium-to-large overall effect. Both interaction effects were statistically significant: task complexity × felt process accountability focus (β = −0.04, *p* = 0.012) and task complexity × felt outcome accountability focus (β = 0.04, *p* = 0.006). The interaction effect accounted for approximately 4 % of this variance (η² = 0.02 each), representing a small but theoretically meaningful contribution within the model.

**Table 2:** Linear mixed-model analysis of missed nursing care by felt accountability focus, task complexity, and their interaction.

	Step 1 Control variables				Step 2 Independent variables			Step 3 Interaction Effect	
Variables	Beta (SE)	p-value	95 % CI	Beta (SE)	p-value	95 % CI	Beta (SE)	p-value	95 % CI
Professional nursing role - Clinical instructor	−0.06	0.473	−0.23, 0.11	−0.07	0.434	−0.24, 0.11	−0.09	0.339	−0.26, 0.09
Professional nursing role –Deputy head nurse	−0.06	0.682	−0.35, 0.23	−0.06	0.689	−0.35, 0.24	−0.05	0.715	−0.35, 0.24
Workload	0.04	0.000	0.03, 0.05	0.03	0.000	0.02, 0.04	0.03	0.000	0.02, 0.04
Felt process accountability focus				−0.01	0.605	−0.04, 0.05	0.13	0.041	−0.22, −0.02
Felt outcome accountability focus				0.01	0.743	−0.07, 0.04	−0.12	0.021	0.01, 0. 26
Task complexity				0.03	0.098	−0.01, 0.07	0.09	0.266	−0.07, 0.24
Felt process accountability focus x Task complexity							−0.04	0.012	0.01, 0.07
Felt outcome accountability focus x Task complexity							0.04	0.006	−0.08, −0.01
Δ−2 log likelihood				14.53	0.002		4.57	0.101	
Residual	0.06 (0.01)			0.06 (0.01)			0.05 (0.00)		
Variance	0.06 (0.01)			0.06 (0.01)			0.06 (0.01)		

**Fig. 2:**
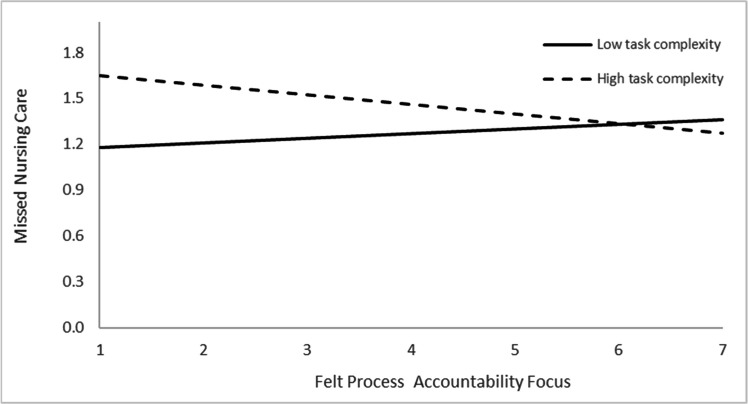
The interaction effect of felt process accountability focus and task complexity on missed nursing care.

**Fig. 3:**
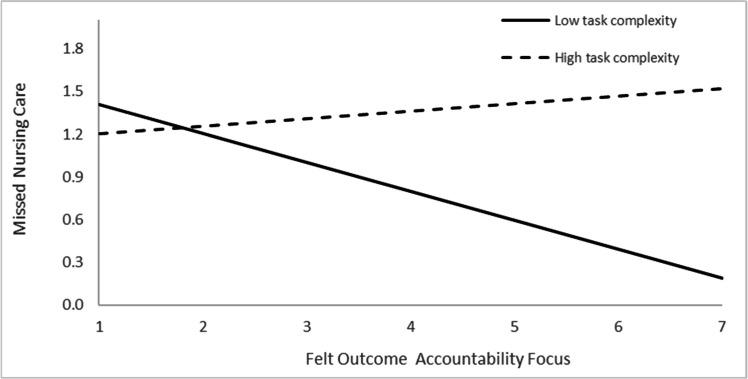
The interaction effect of felt outcome accountability focus and task complexity on missed nursing care.

Fig. 2, Fig. 3 illustrate these interaction effects. As shown in Fig. 2, under high task complexity, nurses with higher levels of felt process accountability focus reported lower frequency of missed nursing care compared to those with lower felt process accountability focus; whereas under low task complexity, felt process accountability was not statistically significantly associated with missed nursing care.

Conversely, Fig. 3 shows that under lower task complexity, nurses with higher felt outcome accountability focus reported lower frequency of missed nursing care compared to those with lower felt outcome accountability focus; whereas under high task complexity, felt outcome accountability was not statistically significantly associated with missed nursing care.

## 4 Discussion

This study is the first to demonstrate in real-world nursing practice that the effect of felt accountability focuses on missed nursing care depends on task complexity. While previous research has largely relied on laboratory simulations or non-clinical samples (Langhe et al., 2011; Sharon et al., 2024; Siegel-Jacobs and Yates, 1996), our findings provide field-based evidence that felt accountability focus alone does not predict performance; rather, its value emerges only in interaction with the cognitive demands of care tasks. Importantly, the results highlight that accountability focus is not merely an abstract organizational construct, but a motivational force genuinely perceived by nurses in their daily practice, shaping how they engage with and respond to clinical demands. By applying the accountability framework (Frink and Klimoski, 2004) together with the resource allocation model (Kanfer and Ackerman, 1989) this study contributes a theoretically grounded explanation of when accountability helps nurses reduce omissions in care and when it does not.

Consistent with Hypothesis 1, no statistically significant effects of felt outcome accountability or felt process accountability on missed nursing care was found. This aligns with meta-analysis (Sharon et al., 2022), which reported inconsistent and weak main effects of accountability focus on performance. The absence of direct effects in our study underscores that accountability cannot be understood as a simple predictor of performance; instead, its impact must be examined as contingent on the work context. This reinforces the person-in-context perspective, which emphasizes the interplay between individual motivation, organizational mechanisms, and task demands in shaping professional behavior (Hall et al., 2017; Hochwarter et al., 2007).

In line with Hypothesis 2a, however, the interaction analyses revealed important conditional effects. Under conditions of high task complexity, nurses who reported higher levels of felt process accountability focus reported fewer missed care events compared with their peers. This finding strengthens the accountability framework, which highlights that being answerable for the process of action promotes deliberate reasoning, fairness, and ethical engagement (Scholten et al., 2007). It also aligns with the resource allocation model, which argues that individuals allocate finite cognitive and emotional resources in response to task demands (Kanfer and Ackerman, 1989). Complex clinical situations require heightened cognitive investment; process accountability focus directs attention to how care is delivered, ensuring that scarce resources are allocated to careful assessment, prioritization, and execution. Furthermore, process accountability likely enhances metacognitive monitoring—nurses’ ongoing awareness and regulation of their own decision processes—which helps detect early signs of potential omissions or errors before they escalate. Moreover, process accountability may reduce psychological strain by providing nurses with a sense of control and procedural fairness (Häusser et al., 2017), which helps sustain attention and emotional stability in demanding situations. By reducing reliance on shortcuts and reinforcing reflective practice, process accountability focus protects care quality in situations where the risk of omission is greatest (Sharon et al., 2024). Together, these mechanisms illustrate how process accountability translates cognitive engagement into behavioral precision, ultimately reducing care omissions in cognitively demanding contexts.

Conversely, in low-complexity tasks, felt outcome accountability focus was associated with fewer missed care events, supporting Hypothesis 2b From a theoretical standpoint, outcome accountability focus provides clear performance anchors and emphasizes efficiency (Hall et al., 2007), which is advantageous when tasks are routine and cognitively undemanding. The resource allocation model explains why this is beneficial: simple tasks consume fewer resources, making it efficient to direct attention toward results without taxing reflective processes (Häusser et al., 2017; Kanfer and Ackerman, 1989). Additionally, in routine contexts, the predictability of outcomes allows accountability metrics to act as reinforcing cues that enhance motivation, consistency, and adherence to standards (Foulkes, 2011). It is possible that under these conditions, outcome accountability activates extrinsic motivational pathways—such as reward sensitivity and goal attainment orientation—thereby strengthening compliance with organizational standards even when intrinsic motivation is low. In this way, outcome accountability focus helps ensure that straightforward nursing activities, such as vital sign monitoring or timely response to call lights, are completed consistently and reliably.

Importantly, not all felt accountability focus-task pairings were effective. Felt process accountability focus in simple tasks and felt outcome accountability focus in complex tasks were not statistically significantly related to reductions in missed care. These null findings are themselves informative. They highlight that accountability mechanisms are not universally beneficial; their utility depends on congruence with task demands. In complex care, outcome accountability focus may inadvertently heighten pressure and shift focus toward results that are partly outside nurses’ control (Hall et al., 2007; Siegel-Jacobs and Yates, 1996). This mismatch likely increases cognitive overload and emotional strain, diverting attention from clinical reasoning and teamwork. In such cases, accountability may be experienced less as a motivational driver and more as a form of evaluative surveillance, which undermines psychological safety and reduces openness to reflection or help-seeking behaviors. Similarly, in simple tasks, process accountability focus may be redundant, offering little incremental value when the task can be completed efficiently without deep reflection (Langhe et al., 2011). This redundancy may even slow performance by introducing unnecessary deliberation, thereby diminishing efficiency without meaningful gains in accuracy. These results caution against the indiscriminate application of accountability structures and underscore the importance of aligning them with the nature of clinical work.

Taken together, these findings suggest that accountability operates as a context-sensitive motivational mechanism rather than a uniform managerial tool (Frink and Klimoski, 2004; Hall et al., 2007). Its effectiveness depends on the cognitive and emotional resources required by the task and the degree of control nurses perceive over outcomes (Häusser et al., 2017). When perceived accountability matches both the cognitive load and the locus of control inherent to the task, it optimizes resource allocation, enhances focus, and supports adaptive behavior (Kanfer and Ackerman, 1989). When perceived accountability focus aligns with task complexity—process accountability for complex care and outcome accountability for routine care—it enhances attention, reasoning, and efficiency. However, when misaligned, accountability can create pressure, cognitive overload, or disengagement, thereby increasing the risk of omissions (Sharon et al., 2024). These mechanisms highlight that accountability is not only a structural demand but also a subjective experience shaped by nurses’ cognitive appraisal of task controllability and organizational expectations. This nuanced understanding highlights the importance of adaptive accountability systems that acknowledge the variability of nursing work and the cognitive and emotional demands inherent in diverse care situations.

### 4.1 Theoretical and practical contributions

Theoretically, this study contributes in two key ways. First, it integrates the accountability framework (Frink and Klimoski, 2004; Hall et al., 2017) and the resource allocation model (Kanfer and Ackerman, 1989) to explain how accountability focus interacts with task demands in nursing practice. By showing that accountability focus’ influence is conditional, the study extends both models and provides empirical evidence for the fit perspective, which posits that organizational mechanisms must align with situational demands to produce optimal outcomes (Häusser et al., 2017). Second, it advances accountability research beyond controlled experiments (Langhe et al., 2011; Sharon et al., 2024) to real-world acute care, where omissions in care have direct implications for both nurses’ and patients’ outcomes (Bayadsi et al., 2025; Chaboyer et al., 2021; Cohen et al., 2024; Lake et al., 2019). This transition from laboratory to clinical settings strengthens the external validity of accountability theory and demonstrates its practical relevance in healthcare.

From a practice standpoint, the findings provide actionable guidance for healthcare leaders and policymakers. Accountability structures should not be designed as “one size fits all.” For routine, low-complexity tasks, outcome-focused accountability may enhance efficiency and ensure that basic care is reliably delivered (Foulkes, 2011). In contrast, for complex, high-stakes clinical situations, process-focused accountability is critical for fostering careful reasoning, adaptive decision-making, and thoroughness (Bail and Grealish, 2016; Nobahar et al., 2023).

Nurse managers should therefore match accountability structures to the cognitive demands of specific tasks by integrating this principle into both training and evaluation systems. Practically, this can be achieved through simulation-based training and structured debriefing sessions that emphasize reflective practice and clinical reasoning rather than solely outcome metrics (Cant and Cooper, 2017). Evaluation and feedback systems could similarly incorporate process indicators, such as adherence to evidence-based protocols or quality of clinical handovers, alongside traditional outcome indicators.

Such targeted strategies would better align accountability frameworks with task demands, reinforcing both care quality and professional development. Beyond ward-level management, the findings also inform performance appraisal systems, workforce development, and nursing education (Cohen et al., 2024; Sharon et al., 2024). Embedding discussions on how different accountability mechanisms shape decision-making under different conditions into nursing curricula could help prepare future nurses for the complex realities of clinical practice.

### 4.2 Strength, limitations, and recommendations for further research

Several strengths reinforce the contribution of this study. Employing a prospective repeated-measures design in real-world nursing encounters is rare in accountability research and allowed us to capture in vivo processes rather than relying on hypothetical scenarios. Including nurses from multiple wards and specialties also increased internal variability, strengthening the robustness of findings.

Nonetheless, limitations should be acknowledged. First, the measure of felt accountability focus relied on nurses’ self-reported perceptions rather than externally imposed or observed mechanisms. However, this is consistent with Hall et al. (2006), who conceptualized accountability as an internalized sense of being answerable, making self-report theoretically appropriate. Prior research also found no statistically significant differences between nurse self-reported and peer-reported missed nursing care (Srulovici and Drach-Zahavy, 2017), supporting its validity. Still, future research should complement self-report with observational or managerial ratings.

Second, task complexity was assessed as a general perception of the shift rather than linked to specific nursing tasks. This limits the precision of the findings, since missed care often occurs at the task level. At the same time, measuring task complexity at the shift level captures how nurses subjectively experience their workload as an integrated whole, reflecting the cumulative burden of multiple overlapping tasks and situational demands. This subjective perspective is not only ecologically valid but also supported by recent evidence showing that perceived workload can be as influential as, and sometimes more predictive than, objective workload in explaining nursing outcomes (Bayadsi et al., 2025). Future studies could build on this approach by incorporating both subjective and objective indicators of task complexity, and by linking them to specific nursing tasks, to provide a more nuanced understanding of how complexity shapes missed care.

Finally, the study was conducted in a single hospital, which may limit its generalizability to other organizational or cultural contexts. Although nurses were recruited from multiple wards and specialties—surgical, rehabilitation, and internal medicine—providing substantial variation in functions, care approaches, and patient populations, hospital-specific factors such as organizational culture, leadership practices, or resource allocation might have influenced the observed patterns. The inclusion of diverse wards staffed by specialized professionals enhances internal variability and strengthens the robustness of the findings. Nevertheless, as these wards are commonly represented in missed nursing care research due to their high prevalence of omissions and impact on patient outcomes, caution is warranted when extending the results to other healthcare settings. Future studies should include multiple hospitals and countries to enable broader validation across diverse organizational and cultural environments.

## 5 Conclusion

This prospective repeated-measures study in real-world nursing encounters provides novel evidence that felt accountability focus influences missed nursing care only in interaction with task complexity. Felt outcome accountability was effective in reducing omissions during simple, routine tasks, whereas felt process accountability was essential in complex, high-stakes care, underscoring the need for accountability frameworks that are flexible and aligned with the cognitive demands of nursing work. These insights advance theoretical understanding of accountability as a conditional mechanism, extend empirical knowledge from laboratory studies into clinical practice, and offer practical guidance for leaders and policymakers seeking to reduce missed care and improve patient safety. Future research should further examine how additional contextual and organizational factors, such as leadership style, team support, and cultures of organizational learning, shape the relationship between focus accountability and performance in complex healthcare environments.

## Funding

This study was supported by the Cheryl Spencer Institute of Nursing Research and the Israel Science Foundation No. 738/21.

## CRediT authorship contribution statement

**Layla Suliman:** Writing – review & editing, Writing – original draft, Project administration, Methodology, Investigation, Formal analysis, Data curation, Conceptualization. **Anat Drach- Zahavy:** Writing – review & editing, Project administration, Methodology, Conceptualization. **Hadass Goldblatt:** Writing – review & editing, Methodology, Conceptualization. **Hanna Admi:** Writing – review & editing, Methodology, Conceptualization. **Ilana Peterfreund:** Writing – review & editing, Methodology, Conceptualization. **Liora Sabah:** Writing – review & editing, Methodology, Conceptualization. **Einav Srulovici:** Writing – review & editing, Project administration, Methodology, Formal analysis, Conceptualization.

## Declaration of competing interest

The authors declare the following financial interests/personal relationships which may be considered as potential competing interests: Anat Drach- Zahavy, Hadass Goldblatt, Hanna Admi, Einav Srulovici reports financial support was provided by The Israel Science Foundation. Layla Suliman reports financial support was provided by University of Haifa The Cheryl Spencer Institute for Nursing Research. If there are other authors, they declare that they have no known competing financial interests or personal relationships that could have appeared to influence the work reported in this paper.
